# Necessity of Prophylactic Extrapleural Chest Tube During Primary Surgical Repair of Esophageal Atresia: A Systematic Review and Meta-Analysis

**DOI:** 10.3389/fped.2022.849992

**Published:** 2022-03-18

**Authors:** Martin Riis Ladefoged, Steven Kwasi Korang, Simone Engmann Hildorf, Jacob Oehlenschlæger, Susanne Poulsen, Magdalena Fossum, Ulrik Lausten-Thomsen

**Affiliations:** ^1^Copenhagen Trial Unit, Department 7812, Centre for Clinical Intervention Research, Copenhagen University Hospital Rigshospitalet, Copenhagen, Denmark; ^2^Department of Clinical Medicine, University of Copenhagen, Copenhagen, Denmark; ^3^Department of Anesthesiology Critical Care Medicine, Childrens Hospital Los Angeles, Los Angeles, CA, United States; ^4^Department of Paediatric Surgery, Copenhagen University Hospital Rigshospitalet, Copenhagen, Denmark; ^5^Department of Neonatology, Copenhagen University Hospital Rigshospitalet, Copenhagen, Denmark; ^6^Department of Women's and Children's Health, Karolinska Institutet, Stockholm, Sweden

**Keywords:** chest tube, neonates, tracheoesophageal fistula, esophageal atresia, pediatric surgery

## Abstract

**Background:**

Esophageal atresia is corrected surgically by anastomosing and recreating esophageal continuity. To allow the removal of excess fluid and air from the anastomosis, a prophylactic and temporary intraoperative chest tube (IOCT) has traditionally been placed in this area during surgery. However, whether the potential benefits of this prophylactic IOCT overweigh the potential harms is unclear.

**Objective:**

To assess the benefits and harms of using a prophylactic IOCT during primary surgical repair of esophageal atresia.

**Data Sources:**

We conducted a systematic review with a meta-analysis. We searched Cochrane Central Register of Controlled Trials (2021, Issue 12), MEDLINE Ovid, Embase Ovid, CINAHL, and Science Citation Index Expanded and Conference Proceedings Citation Index—(Web of Science). Search was performed from inception until December 3rd, 2021.

**Study Selection:**

Randomized clinical trials (RCT) assessing the effect of a prophylactic IOCT during primary surgical repair of esophageal atresia and observational studies identified during our searches for RCT.

**Data Extraction and Synthesis:**

Two independent reviewers screened studies and performed data extraction. The certainty of the evidence was assessed by GRADE and ROBINS-I.

**PROSPERO Registration:**

A protocol for this review has been registered on PROSPERO (CRD42021257834).

**Results:**

We included three RCTs randomizing 162 neonates, all at overall “some risk of bias.” The studies compared the placement of an IOCT vs. none. The meta-analysis did not identify any significant effect of profylacitic IOCT, as confidence intervals were compatible with no effect, but the analyses suggests that the placement of an IOCT might lead to an increase in all-cause mortality (RR 1.66, 95% CI 0.76–3.65; three trials), serious adverse events (RR 1.08, 95% CI 0.58–2.00; three trials), intervention-requiring pneumothorax (RR 1.65, 95% CI 0.28–9.50; two trials), and anastomosis leakage (RR 1.66, 95% CI 0.63–4.40). None of our included studies assessed esophageal stricture or pain. Certainty of evidence was very low for all outcomes.

**Conclusions:**

Evidence from RCTs does not support the routine use of a prophylactic IOCT during primary surgical repair of esophageal atresia.

## Introduction

Esophageal atresia refers to a group of congenital anomalies in which the continuity of the esophagus is interrupted (1). Tracheoesophageal anomalies are divided into subtypes depending on anatomy and the most prominent (85%) subtype has a tracheoesophageal fistula to the distal esophageal segment (1). The prevalence of esophageal atresia varies according to country and time period (2–8). Observational studies from 1981 to 2018 have estimated the prevalence to span from 0.88 to 4.55 per 10,000 births in China and in Germany, respectively. Recent European studies suggest that the prevalence is relatively stable over time (9–11) and that males are most affected with a male:female ratio of 1:0.74 (9).

At birth, the neonate presents with typical drooling of saliva, inability to swallow, choking, coughing, cyanotic attacks, and distended abdomen if the subtype involves a fistula to the trachea (11). The diagnosis is confirmed by the inability to pass a feeding tube into the stomach (11, 12) and a plain X-ray showing the non-progression of the feeding tube located in a blind-ending pouch (11, 12). Prenatal diagnostics having improved from 26 to 36% during the last 30 years (9). Postnatal diagnosis occurs on the first day after birth in 83% of cases, the remaining 15% of cases within the first week, and only in 1.2% after the first week (9).

Most cases seem to occur sporadically, therefore the etiology is likely to be multifactorial involving multiple genes and complex gene-environment interactions (13, 14). Despite observational studies suggesting various maternal risk factors (10, 13, 15), the exact etiology is still unclear (13, 14). Since esophageal atresia is an early organogenesis defect, associated anomalies are frequency found (9, 16–19). Isolated esophageal atresia occurs in ~45–53% of the cases, whereas 32–47% have multiple anomalies, and 24–25% have an association or a syndrome, the most common being VACTERL association occurring in ~10% (9, 16–21). Among the most common associated anomalies are congenital heart defects (23–29% of cases), other gastrointestinal anomalies (16–21%), urinary tract anomalies (15–16%), and limb anomalies (13–14%) (9, 16–19).

Left untreated, the condition is fatal due to starvation, infection, and respiratory complications and survival therefore relies on early surgical correction (1, 11). The surgery aims to reconstruct the continuity of the esophagus and eliminate any possible fistulae (11, 14), which can be done either as a transpleural thorascopical procedure, or as open surgery, most commonly extrapleural (11, 22).

The mortality rate in isolated esophageal atresia range from 4.3 to 8.1% (7, 8, 17, 23–25), but varies with the type of atresia (with higher mortality and morbidity in the long gap esophageal atresia presentation), and mortality increases furthermore in case of prematurity and/or low birth weight, and with the presence of associated abnormalities, notably major cardiac and chromosomal anomalies (2, 6, 9, 26, 27). Even after hospital discharge, the children have increased mortality with post-discharge mortality is primarily due to respiratory compromise, including sudden infant death, aspiration, tracheomalacia, and reactive airway disease (23, 28, 29).

The most common postoperative complications are anastomosis leakage, fistula recurrence, anastomotic strictures, respiratory complications, and infections (25, 30–40). Anastomotic leakage is one of the most common serious complications occurring in about 5–17% of cases (25, 34, 35). Leakage into the mediastinum result mainly from anastomotic tension (particularly in cases with increased gap length) leading to ischemia of the esophageal ends, particularly in the small, friable lower segment and sub-optimal surgical technique; sepsis and even use of prosthetic materials can contribute (36–38). Major leaks are uncommon and tend to present with acute deterioration associated with pneumothorax and sepsis, and may require emergency decompression with placement of a postoperative chest tube (35, 39, 40). Most leaks heal spontaneously given proper drainage and antibiotics and only few require surgical intervention (35, 39, 40).

Long-term complications include strictures of the anastomotic region [incidence 25–75% (25, 34, 41, 42)], gastroesophageal reflux [incidence 22–63% (43)], esophagitis, tracheomalacia, feeding difficulties [incidence up to 80% (30, 44)], pulmonary symptoms, and developmental challenges (30–33, 45). These long-term complications have an impact on quality of life (28) in both patients and parents, especially in the case of tracheal and esophageal complications (6, 31, 46–50).

During the esophageal repair a prophylactic intraoperative chest tube (IOCT) has traditionally been placed close to the anastomosis to drain access fluid and air through a one-way system (51, 52). The routine use of prophylactic IOCTs is now debated (11, 39, 40, 52) and at the European Reference Network for rare Inherited and Congenital Anomalies (ERNICA) consensus conference, no consensus was found with only 21.4% of the members voting for the use of IOCTs (22). However, IOCTs are still used and reported as common as in 54% of the cases in the UK (53), 57% in Belgium (54), and 69% in an international survey, respectively (55).

IOCTs are not without drawback as they can cause insertion site infection (56) and when improperly placed, the tube can cause disruption of the site of anastomosis or penetration of proximal myotomy (57). IOCTs may also cause considerable postoperative pain, which would decrease inspiratory effort tand need for administration of more opoids, both leading to secondary effects such as atelectasis and pneumonia (58). Importantly, in some cases, IOCTs are insufficient to drain major leaks, necessitating the placement of a new chest tube (39, 40).

Whether the potential benefits of the prophylactic IOCT overweigh the potential harms is therefore unclear (22). Accordingly, the objective of this study was to examine the benefits and harms of prophylactic IOCT during primary surgical repair of esophageal atresia.

## Methods

We conducted a systematic review of the existing literature according to the Preferred Reporting Items for Systematic Reviews and Meta-Analysis guidelines (PRISMA) and the Cochrane Handbook for Systematic Reviews of Intervention (59, 60). The predefined methodology, and method for this review in general, is described in our protocol, registered in June 2021 (61).

### Eligibility Criteria

We searched for RCTs assessing the effect of a prophylactic IOCT during primary surgical repair of esophageal atresia and related observational studies identified during our searches for RCTs.

### Search and Study Selection

We searched the Cochrane Central Register of Controlled Trials, MEDLINE Ovid, Embase Ovid, CINAHL, and Science Citation Index Expanded and Conference Proceedings Citation Index—(Web of Science). The search strategy was developed by an information specialist from the Cochrane Hepato-Biliary Group. The search strategy can be found in Appendix 1.

Studies were included irrespectively of publication type, publications status, and language. Two independent reviewers (MRL and SKK) screened and found relevant studies, performed data-extraction using an EXCEL data extraction sheet, and systematically checked risks of bias. We planned to contact trial authors if relevant data were unclear or missing. A description of the data collection process can be found in our protocol.

### Risk of Bias Assessment

We followed the Cochrane Handbook for Systematic Reviews of Interventions to examine the risk of bias (62), including the ROBIN-I tool for non-randomized studies (63). Two authors, MRL and SKK, independently assessed the risk of bias in the included trials. In case of disagreements, a third author (ULT) would arbitrate.

### Outcomes and Subgroup Analyses

The primary outcomes were: (1) all-cause mortality, (2) serious adverse events, and (3) pneumothorax - requiring intervention. Secondary outcomes were: (1) sepsis or mediastinitis, (2) anastomosis leakage, (3) esophageal stricture, and (4) pain (measured by any valid score). For every relevant outcome, the risk ratios (RRs) were calculated with a 95% confidence interval (CI).

### Data Synthesis

We pooled the data from relevant studies that were estimated to be clinically homogeneous using the Review Manager 5.4.1 software. If more than one study provides usable data in any single comparison, we performed a meta-analysis. We used RR for dichotomous outcomes, and by utilizing the fixed-effect (Mantel-Haenszel model).

## Results

**Figure 1:**
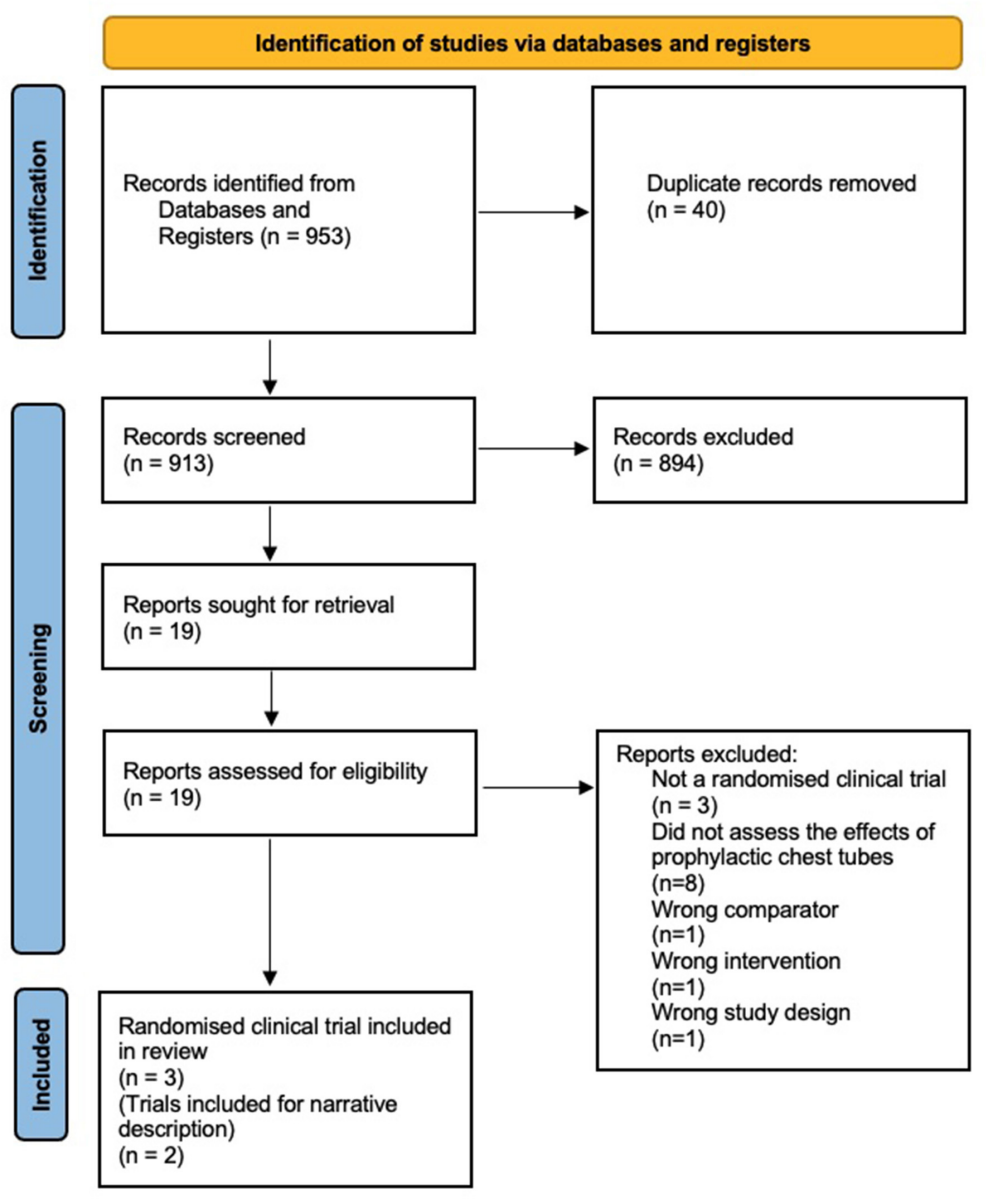
PRISMA flowchart.

A systematic search done December 3rd, 2021, identified a total of 953 records from databases and registers. A total of 894 were excluded based on the title and abstract. We assessed 19 full-text original articles, of which following studies were included: three RCTs (64–66) and two case-control studies (51, 52) for narrative description in the discussion. See Figure 1: PRISMA flowchart and Table 1: Table of excluded studies regarding details on inclusion and exclusion of the studies.

**Table 1:** Table of excluded studies.

**Study id**	**Reason for exclusion**	**The authors's conclusion on IOCT (if any)**
Brohi et al. (67)	Not a randomized clinical trial	NA
Castilloux et al. (68)	Did not assess the effects of prophylactic chest tubes	NA
Donoso et al. (69)	Did not assess the effects of prophylactic chest tubes	NA
Esteves et al. (70)	Did not assess the effects of prophylactic chest tubes	NA
Fasting and Winther (71)	Did not assess the effects of prophylactic chest tubes	NA
Grebe et al. (72)	Wrong intervention	NA
Johnson and Wright (57)	Wrong study design	An IOCT can perforate esophagus after primary repair.
Kay and Shaw (73)	Wrong comparator	An IOCT may not be necessary.
McCallion et al. (40)	Not a randomized clinical trial	IOCT unable to drain major leaks sufficiently, requiring placement of an additional drain.
Paramalingam et al. (74)	Not a randomized clinical trial	Drain appears not to be needed in all cases.
Vazquez et al. (75)	Did not assess the effects of prophylactic chest tubes	NA
Vercauteren et al. (76) Vol 8	Did not assess the effects of prophylactic chest tubes	NA
Zhang et al. (77)	Did not assess the effects of prophylactic chest tubes	NA
Zhang et al. (78)	Did not assess the effects of prophylactic chest tubes	NA

*NA, not applicable*.

### Included Trials

**Figure 2:**
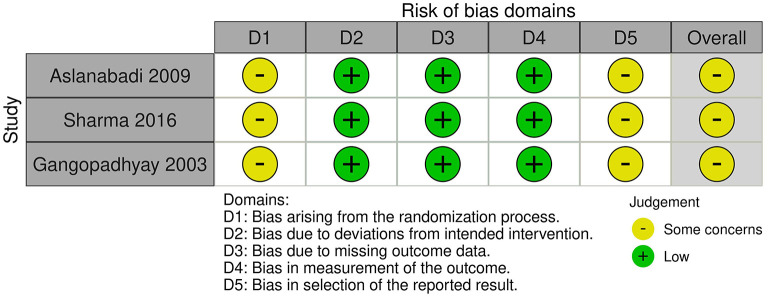
Risk of bias assessment.

We identified and included three RCTs randomizing a total of 162 neonates with esophageal atresia and distal tracheoesophageal fistula into intervention and control group. The trials compared mortality, serious adverse events, intervention-requiring pneumothorax, and anastomosis leakage (see Table 2: summary of findings). The risk of bias assessment is shown in Figure 2. All trials were assessed to be at overall “some concerns” for risk of bias. None of the trials adequately describe the randomization process or referred to a publish protocol.

**Table 2:** Summary of findings, randomized clinical trials.

**Use of prophylactic chest tubes vs. control**						
**Population:** Neonates with esophageal atresia.						
**Intervention:** Prophylactic chest tube in primary surgical repair.						
**Comparison**: Control (no prophylactic chest tube).						
**Outcomes**	Illustrative comparative risks^*^(95% CI)		**Relative effect (95% CI)**	**No of participants (No of studies)**	**Quality of the evidence (GRADE)**	**Comments**
	**Assumed risk (controls)**	**Corresponding risk (chest tube)**				
	**Study population**					
**All-cause mortality**						
Maximum follow-up	109 per 1,000	182 per 1,000 (83–398)	RR 1.66 (0.76, 3.65)	162 (3)	⊕⊖⊖⊖ **Very low**	OIS 5822 (alpha 5%, beta 20%, RR 0.8 and Pc 10.9%) Downgraded one level due to serious risk of bias and two levels due to very serious imprecision.
**Serious adverse events**						
Maximum follow-up	250 per 1,000	270 per 1,000 (145–500)	RR 1.08 (0.58, 2.00)	162 (3)	⊕⊖⊖⊖ **Very low**	OIS 2188 (alpha 5%, beta 20%, RR 0.8 and Pc 25%) The adverse events reported were respiratory complications. Downgraded one level due to serious risk of bias and two levels due to very serious imprecision.
**Intervention-requiring pneumothorax**						
Maximum follow-up	28 per 1,000	47 per 1,000 (8–271)	RR 1.65 (0.28, 9.50)	112 (2)	⊕⊖⊖⊖ **Very low**	OIS 24124 (alpha 5%, beta 20%, RR 0.8 and Pc 25%)
**Sepsis or mediastinitis**						
Maximum follow-up	NA	NA	RR 3.00 (0.14, 64.26)	16 (1)	⊕⊖⊖⊖ **Very low**	
**Anastomosis leakage**						
Maximum follow-up	89 per 1,000	148 per 1,000 (56–393)	RR 1.66 (0.63, 4.40)	162 (3)	⊕⊖⊖⊖ **Very low**	OIS 7240 (alpha 5%, beta 20%, RR 0.8 and Pc 8.9%) Downgraded one level due to serious risk of bias and two levels due to very serious imprecision.
**Esophageal stricture**						
Maximum follow-up	NA	NA				

* *The basis for the assumed risk (e.g., the median control group risk across studies) is provided in footnotes. The corresponding risk (and its 95% confidence interval) is based on the assumed risk in the comparison group and the relative effect of the intervention (and its 95% CI)*.

*CI, Confidence interval; Pc, Proportion in control group with outcome; RR, Risk ratio; NA, Not applicable*.

*GRADE Working Group grades of evidence*.

*High quality: Further research is very unlikely to change our confidence in the estimate of effect*.

*Moderate quality: Further research is likely to have an important impact on our confidence in the estimate of effect and may change the estimate*.

*Low quality: Further research is very likely to have an important impact on our confidence in the estimate of effect and is likely to change the estimate*.

*Very low quality: We are very uncertain about the estimate*.

### Effects of Interventions

#### Primary Outcomes

##### All-Cause Mortality

**Figure 3:**
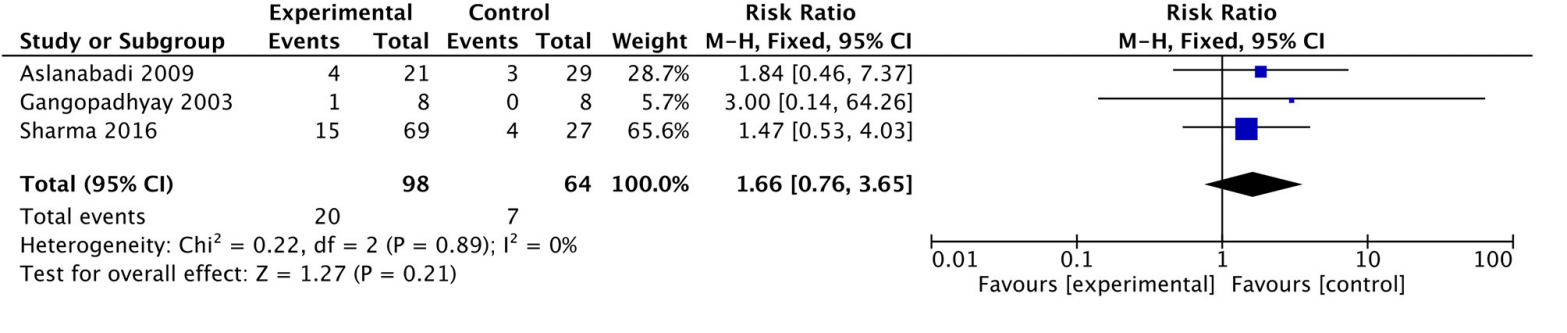
Forest plot for all-cause mortality.

A meta-analysis of three trials, randomizing 162 participants, showed that an IOCT might result in an increased risk of mortality compared to neonates undergoing surgery without an IOCT, but the confidence interval was compatible with no effect [RR 1.66, 95% CI 0.76–3.65; *P* = 0.21; 2.8% of optimal information size (OIS); very low certainty of evidence; Figure 3].

##### Proportion of Participants With One or More Serious Adverse Events

**Figure 4:**
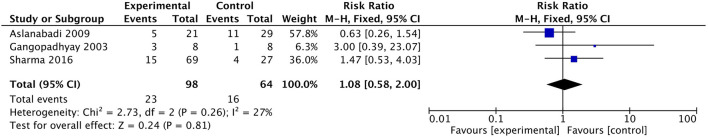
Forest plot for serious adverse events.

A meta-analysis of three trials, randomizing 162 participants, showed that an IOCT might result in an increased risk of having a serious adverse event compared with neonates with esophageal atresia undergoing surgery without an IOCT, but the confidence interval was compatible with no effect (RR 1.08, 95% CI 0.58–2.00; *P* = 0.81; 7.4% of OIS; very low certainty of evidence; Figure 4).

The serious adverse effects assessed in the trials were respiratory complications including respiratory distress, pneumonia, pneumothorax, lung collapse, and apnea as well as mortality.

##### Proportion of Participants With an Intervention-Requiring Pneumothorax

**Figure 5:**
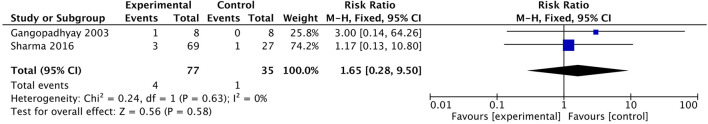
Forest plot for intervention-requiring pneumothorax.

A meta-analysis of the two trials, randomizing 112 participants, showed that an IOCT might result in an increased risk of having an intervention-requiring pneumothorax compared with neonates with esophageal atresia undergoing surgery without an IOCT, but the confidence interval was compatible with no effect (RR 1.65, 95% CI 0.28–9.50; *P* = 0.58; 0.46% of OIS; very low certainty of evidence; Figure 5).

#### Secondary Outcomes

##### Participants With Sepsis or Mediastinitis

One included trial (65), reporting sepsis, showed that an IOCT might result in an increased risk of having sepsis compared with neonates with esophageal atresia undergoing surgery without an IOCT, but the confidence interval was compatible with no effect (RR 3.00, 95% CI 0.14–64.26).

##### Participants With Anastomosis Leakage

**Figure 6:**
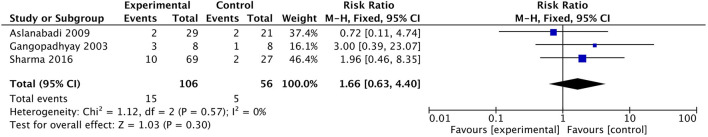
Forest plot for anastomosis leakage.

Three trials, randomizing 162 participants, showed that an IOCT might result in an increased risk of anastomosis leakages compared with neonates with esophageal atresia undergoing surgery without an IOCT, but the confidence interval was compatible with no effect (RR 1.66, 95% CI 0.63–4.40; *P* = 0.30; 2.24 % of OIS; very low certainty of evidence; Figure 6).

##### Participants With Esophageal Stricture

None of the included trials reported on esophageal stricture.

##### Pain (Measured by Any Valid Score)

None of the included studies did a measurement of pain.

## Discussion

### Summary of Main Findings

We identified and included three RCTs randomizing a total of 162 neonates with esophageal atresia and distal tracheoesophageal fistula into intervention and control group. The trials compared mortality, serious adverse events, intervention-requiring pneumothorax, and anastomosis leakage.

We found no evidence of a beneficial effect of placing a prophylactic IOCT during primary surgical repair from neither of the included studies. The evidence from RCTs shows potential harm when assessing all-cause mortality and serious adverse events, but the results were very uncertain. All studies were assessed to be at overall “some concerns” for risk of bias. The risk of bias assessment is shown in Figure 2. The statistical heterogeneity was low for all our meta-analyses. It was not possible to assess the preplanned subgroups regarding esophageal stricture and pain due to the lack of relevant data.

Two observational studies (51, 52) seem to support the overall results from the three RCTs in terms of mortality, serious adverse events, and anastomosis leakage that found no beneficial effect of placing a prophylactic IOCT. Furthermore, observational data from Nquyen et al. (51) suggest that the placement of a prophylactic IOCT may increase the risk of various complications such as an increase in the risk of developing esophageal stricture. These observational studies were assessed by ROBINS-I to be at overall serious (51) and critical risk of bias (52) and should therefore be interpreted with caution. Finally, we identified but excluded for various reasons (see Table 1) an additional 4 studies, no of which were in favor of routine ICOT (see Table 1).

### Strengths and Limitations

This review draws strengths from the strict methodology, including following a protocol registered before the literature search began, systemically assessing for risk of bias, and adhering to all recommendations from the Cochrane Collaboration, including the use of ROBINS-I. The search strategy was developed by an information specialist from the Cochrane Hepato-Biliary Group. Our study also differs from a recent review by Anand et al. (79) on the topic by adding GRADE assessment of the included studies and abstaining from mixing RCTs with observational studies in the meta-analyses. In Anand et al., the meta-analysis included a mix of extrapleural and transpleural repair (51, 52) and an observation study, where some of the participants received IOCTs by a non-prophylactic indication (74). Although the overall conclusions in the present study are fairly similar to the study by Anand et al., inclusion of non-randomized studies with their inherently different study designs in a meta-analysis may severely compromise the validity of their results, as their lack of randomization makes them highly at risk for confounding bias resulting in an imbalance in prognostic factors associated with the outcome (80).

We only identified three RCTs, systematically comparing the intervention with an IOCT to no IOCT in 162 neonates undergoing primary repair for esophageal atresia. None of our meta-analyses reached the optimal information size. In addition to evaluate overall improvement in treatment techniques and clinical outcomes, future trials should also assess pain and esophageal stricture as this would be an important outcome for the children and parents. Importantly, the associated malformations and genetic aberrations often found in esophageal atresia and the difference in exact anatomical presentation [with or without fistula(e)] make this a relatively heterogeneous pathology. The patients included in these RCTs all presented with distal tracheoesophageal fistula, but with various details on pre-surgical gap length and on associated malformations; further complicating the comparison between studies and the transferability of the conclusion to other patient subgroups.

Esophageal atresia is a relatively rare condition; particularly considering the numerous subtypes with various possible anatomical presentations and associated malformation. Rare diseases pose challenges to methodology when designing RCTs that are adequately powered to draw definitive conclusions, as small patient sample sizes are statistically vulnerable to small deviations in the observed number of outcomes (81). Innovative clinical trial methods minimizing sample size requirements (82) and optimal research infrastructure (83), possibly through international collaborations, may improve future productivity of robust research in esophageal atresia.

## Conclusion

We did not identify any studies advocating for the use of prophylactic IOCTs. Based on the limited amount of research on this topic and results from the included studies, we did not find sufficient evidence to support or discontinue the routine use of prophylactic IOCTs for neonates undergoing surgical repair of esophageal atresia, as all confidence intervals were compatible with no effect. Further trials, ideally multicentric, are warranted to explore the effects of the prophylactic IOCT for neonates undergoing surgical repair of esophageal atresia. Importantly, future trials should adhere to SPIRIT guidelines (84).

## Data Availability Statement

The original contributions presented in the study are included in the article/supplementary material, further inquiries can be directed to the corresponding author.

## Author Contributions

ML drafted the protocol and review, extracted data, co-ordinated the review, analyzed the data, and revised the review. SK drafted and revised the protocol, extracted data, analyzed the data, interpreted the data, commented on, revised the review, interpreted the data, provided supervision, and provided a methodological and statistical expertise. SH revised and commented on the protocol and review and provided supervision. JO and MF revised and commented on the protocol and review and provided clinical expertise. SP revised and commented on the review. UL-T drafted the protocol, conceived and designed the review, revised the protocol, commented on, revised the review, provided supervision, and clinical expertise. All authors contributed to the article and approved the submitted version.

## Conflict of Interest

The authors declare that the research was conducted in the absence of any commercial or financial relationships that could be construed as a potential conflict of interest.

## Publisher's Note

All claims expressed in this article are solely those of the authors and do not necessarily represent those of their affiliated organizations, or those of the publisher, the editors and the reviewers. Any product that may be evaluated in this article, or claim that may be made by its manufacturer, is not guaranteed or endorsed by the publisher.

## Appendix 1

**Search Strategies (search performed 3**^rd^ **of December 2021)**

Cochrane Central Register of Controlled Trials [*via* Ovid

Evidence-Based Medicine Reviews Database (EBMR)]

**Table:**

#1	MeSH descriptor: [Esophageal Atresia] explode all trees
#2	MeSH descriptor: [Esophagus] explode all trees
#3	(esophag* or oesophag*)
#4	(artresia* or atretic*)
#5	#1 or [(#2 or #3) and #4]
#6	MeSH descriptor: [Chest Tubes] explode all trees
#7	(chest tube* or catheter* or drain* or intubat* or artificial respirat* or suction* or IOCT*)
#8	#6 or #7
#9	#5 and #8
MEDLINE Ovid	
1.	exp Esophageal Atresia/
2.	exp Esophagus/
3.	(esophag* or oesophag*).tw,kw.
4.	(artresia* or atretic*).tw,kw.
5.	1 or [(2 or 3) and 4]
6.	exp Chest Tubes/
7.	(chest tube* or catheter* or drain* or intubat* or artificial respirat* or suction* or IOCT*).mp. [mp=title, abstract, original title, name of substance word, subject heading word, floating sub-heading word, keyword heading word, organism supplementary concept word, protocol supplementary concept word, rare disease supplementary concept word, unique identifier, synonyms]
8.	6 or 7
9.	5 and 8
Embase Ovid	
1.	exp esophagus atresia/
2.	exp esophagus/
3.	(esophag* or oesophag*).tw,kw.
4.	(artresia* or atretic*).tw,kw.
5.	1 or [(2 or 3) and 4]
6.	exp chest tube/
7.	(chest tube* or catheter* or drain* or intubat* or artificial respirat* or suction* or IOCT*).mp. [mp=title, abstract, heading word, drug trade name, original title, device manufacturer, drug manufacturer, device trade name, keyword, floating subheading word, candidate term word]
8.	6 or 7
9.	5 and 8
CINAHL	
S9	S5 AND S8
S8	S6 OR S7
S7	TX (chest tube* or catheter* or drain* or intubat* or artificial respirat* or suction* or IOCT*)
S6	MH chest tubes
S5	S1 or [(S2 or S3) and S4]
S4	TX (artresia* or atretic*)
S3	TX (esophag* or oesophag*)
S2	MH Esophagus
S1	MH Esophageal Atresia
Science Citation Index Expanded and Conference Proceedings Citation Index – (Web of Science)	
#3	#2 AND #1
#2	TS = (chest tube* or catheter* or drain* or intubat* or artificial respirat* or suction* or IOCT*)
#1	TS = [(esophag* or oesophag*) and (artresia* or atretic*)]
